# First molecular detection of *Entamoeba gingivalis* subtypes in individuals from Turkey

**DOI:** 10.1093/femspd/ftad017

**Published:** 2023-07-13

**Authors:** Serra Örsten, Cem Şahin, Engin Yılmaz, Yakut Akyön

**Affiliations:** Hacettepe University, Vocational School of Health Services, Adnan Saygun Street, Ankara, Turkey; Hacettepe University, Vocational School of Health Services, Adnan Saygun Street, Ankara, Turkey; Hacettepe University, Hacettepe Beytepe Hospital, Ankara, Turkey; Hacettepe University, Faculty of Medicine, Department of Medical Biology, Ankara, Turkey; Hacettepe University, Faculty of Medicine, Department of Medical Microbiology, Ankara, Turkey

**Keywords:** *Entamoeba gingivalis*, subtypes, ST1, ST2-kamaktli, Turkey

## Abstract

*Entamoeba gingivalis* is a parasitic protozoan that colonizes the human oral cavity and there are two subtypes (ST1 and ST2) that have been identified to date. However, there are no reports on the molecular detection or characterization of *E. gingivalis* in Turkey. The objective of this study was to detect the presence of *E. gingivalis* in Turkish healthy individuals and those with periodontal disease and to subtype the isolates using molecular techniques. Samples from the oral cavity of 94 individuals were taken and the presence of *E. gingivalis* was determined by PCR using primers for SsrRNA and the amplicons were then confirmed by DNA sequencing. Each participant completed a questionnaire that included demographic data, habits and lifestyle, as well as health status. The presence of *E. gingivalis* was detected in a total of 19 samples (11 patients and eight healthy individuals). Molecular characterization determined that 12 samples belonged to ST1 and seven samples belonged to ST2. The presence of *E. gingivalis* was higher in patients with periodontal disease than in healthy individuals, and this association was statistically significant (*P* < .05). This study constitutes the first report of molecular detection and subtyping of *E. gingivalis* in Turkey.

## Teaser

Genetic characterization of *Entamoeba gingivalis*.

## Introduction

The oral cavity has the second largest number and variety of microorganisms in the body, housing millions of microorganisms that form the oral microbiota (Zhang et al. 2018). These microorganisms form biofilms (dental plaque) and coexist in symbiotic harmony with the host, but when the microbial equilibrium is broken (dysbiosis) different oral diseases such as dental caries and periodontal disease can occur (Dewhirst et al. 2010, Lourenço et al. 2014, Lamont et al. 2018, Yaseen et al. 2021, Martin-Garcia et al. 2022, Santos and Roldán 2023). Bacteria are the most abundant microorganisms (For example: *Streptococcus* spp., *Gemella* spp., *Rothia* spp., *Neisseria* spp., *Haemophilus* spp., *Prevotella* spp., and *Veillonela* spp.) in oral microbiota in healthy individuals (Takeshita et al. 2016, Peters et al. 2017, Negrini et al. 2021). In addition to bacteria, the oral cavity can also harbour protozoa such as *Entamoeba gingivalis* and *Trichomonas tenax*, which received less attention in periodontal studies (Deo and Deshmukh 2019). However, there have been a some studies investigating the colonization of these parasites in the oral cavity of both healthy individuals and those with periodontal disease, yielding variable results (Bonner et al. 2014, Hassan et al. 2019, Trim et al. 2011, Yazar et al. 2016, Yaseen et al. 2021).

*Entamoeba gingivalis* is a protozoan microorganism that is mostly found in the human oral cavity and it has also been detected in the genitourinary tract (Bonner et al. 2014). Transmission occurs through contaminated food or oral equipment, mouth droplets, and kissing (Stensvold et al. 2021). *Entamoeba gingivalis* has a cosmopolitan distribution with a worldwide prevalence of 37% (Badri et al. 2021). Some studies proposed an association between *E. gingivalis* and periodontal diseases (Garcia et al. 2018b, Badri et al. 2021), and in a metagenomic study it was shown that RNA levels of *E. gingivalis* was elevated and the microbial diversity was reduced in the inflamed areas of the mouth (Deng et al. 2017, Bao et al. 2020, Badri et al. 2021).

To date, there are phylogenetically close two subtypes of *E. gingivalis* that have been identified as ST1 and ST2 (García et al. 2018a). These subtypes are suggested to display different patterns of infectious behavior (Garcia et al. 2018b). These subtypes share the same ecological niche in the oral cavity, therefore, it is hard to separate their particular demographic, geographic, or clinical features with few studies done so far.

During the last few decades, many studies have been published on the investigation of the presence of *E. gingivalis* among healthy individuals and patients with several dental diseases with variable results (Abualqomsaan et al. 2010, Ghabanchi et al. 2010, Özçelık et al. 2010, Trim et al. 2011, Bonner et al. 2014). Different or discrepant results may be related to the use of different approaches for the detection of *E. gingivalis*, the selection of different groups of patients, and the genetic diversity that, until now, is still somewhat unknown (García et al. 2018a, b). In Turkey there are not many studies on *E. gingivalis* and the genetic variation has to be explored (Abualqomsaan et al. 2010, Özçelık et al. 2010, Yazar et al. 2016). The objective of this study was to detect the presence of *E. gingivalis* in both healthy individuals and patients with dental diseases by using molecular techniques. Additionally, the study aimed to perform molecular characterization of the identified *E. gingivalis* isolates, which had not previously been done in Turkey.

## Materials and methods

### Study subjects and sample collection

Consecutive patients without any systemic disease (*n* = 94), aged between 18 and 90 years, consulting in the dental clinic at Hacettepe University Beytepe Hospital (Turkey) were selected for this study. Ethical approval for the study was granted by the ethics committee of Hacettepe University, Ankara, Turkey (GO 21/241). Sample collection was carried out between February 2021 and January 2022. From those who have not received antimicrobial therapy in the past 6 months were included in the study. The samples were taken from these patients’ mouths with a sterile disposable cotton swab. The swab was circulated within the gingival pockets, mucosal surfaces, and tooth surface to increase the possibility of *E. gingivalis* detection.

Each participant was asked to fill out a questionnaire that included demographic, education, and income-related questions, as well as questions concerning knowledge about some lifestyle habits such as tap water usage, shared toothbrush usage, mouthwash usage, and smoking. In addition, in order to determine the general health status of the participants, the presence of chronic disease and regular drug usage history was also included in the questionnaire.

### DNA extraction and amplification

Initially, clinical samples in sterile saline were centrifuged at 1200 × *g* for 15 min in order to precipitate all the biological material. Genomic DNA was extracted from clinical samples using the DNA extraction Kit (GeneAll Biotechnology, Korea) according to the manufacturer’s instructions. The concentration and purity of all the obtained DNA was measured by FLUOstar Omega Microplate Reader (BMG LABTECH) using LVis plate. In this study, to identify the subtypes as ST1 and ST2, a nested PCR was performed using the following primer sets Entam1/Entam2 and GEgFST1/GEgFST2- EgST1/2-R. The primer sequences used are given in Table 1. For all PCR reactions, previously reported conditions were applied for the time and temperature (Verweij et al. 2001, Garcia et al. 2018b). Amplicons were visualized under ultraviolet light following electrophoresis on 1.5% (w/v) agarose gel and products seen in the approximately 350 base-pair (bp) band size were considered positive.

**Table 1.** Primers used in study.

Primer	Sequence
Entam1	5′-GTTGATCCTGCCAGTATTATATG-3′
Entam2	5′-CACTATTGGAGCTGGAATTAC-3′
GEgFST1	5′-GAGACGATCCTGTTCTATTAC-3′
GEgFST2	5′GAGACAATCCCAGTTGTTTGTAC3′
EgST1/2-R	5′-ACTATGTACGTTCGTTCATTCC-3′

### Sequence data analyses

Obtained sequence chromatograms were examined using FinchTV viewer (Geospiza, Seattle, WA, USA) and determined the quality of generated nucleotide sequences. Obtained sequences in this study that are present in the NCBI database were compared using the BLAST algorithm (http://www.ncbi.nlm.nih.gov/BLAST/). Sequence data analysis was interpreted as previously described (Boufana et al. 2014). In a few words, data alignment was performed in Mega version 7 and the phylogenetic tree was constructed using ClustalX (Larkin et al. 2007, Tamura et al. 2011). Additionally, Hapview programme was used for generating haplotype networks (Salzburger et al. 2011).

### Statistical analysis

Statistical analysis was performed using IBM SPSS Statistics Version 20 (SPSS Inc., Chicago, IL, USA). The chi-square test was used for group comparisons of categorical data.

## Results

### Demographic and clinical characteristics of study groups

Of the 94 participants, 36 were classified as patients and the rest were considered healthy controls. Most of the participants were female (69.1%, 65/94). The age range of the participants was between 18 and 66 years, and the mean age was 30. Considering the educational status of the participants, most of them (73%, 69/94) were found to have a university and/or higher education degree. Most of the participants (72%, 68/94) were found to have middle/high income; some of them (13.8%, 13/94) had chronic diseases. The majority of the participants (69%, 65/94) indicated not using tobacco, while 87.2% (82/94) reported using spring water. Additionally, most of the participants (85.1%, 80/94) stated that they did not use mouthwash.

### Detection and molecular characterization of *E. gingivalis*

**Figure 1.**
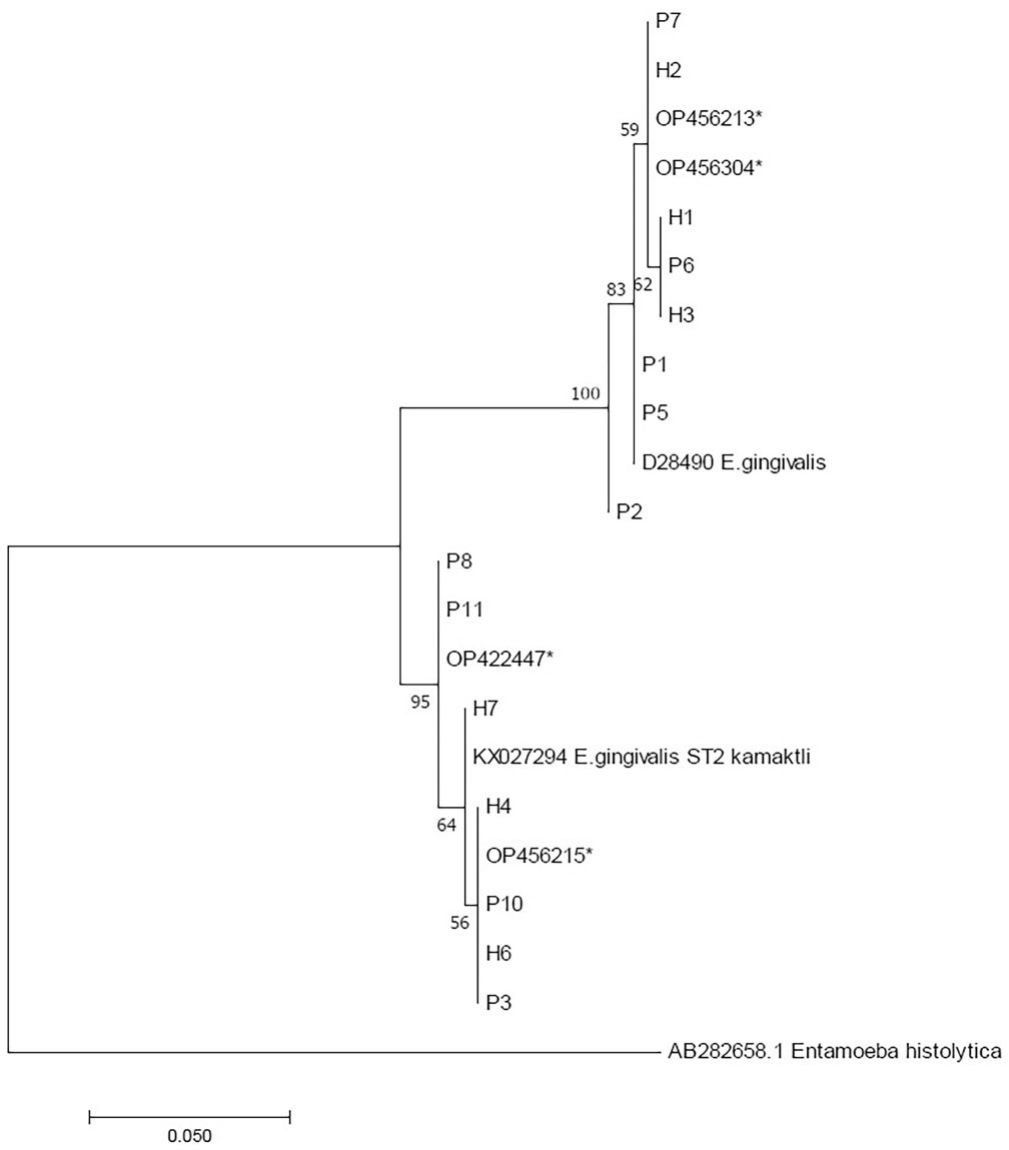
Maximum-likelihood phylogenetic analysis of small subunit ribosomal RNA partial sequences. The obtained sequences in this study and some selected reference sequences (AB282658 *Entamoeba histolytica*, D28490 *E. gingivalis*, KX027294 *E. gingivalis* ST2 kamaktli) were used to conduct a phylogenetic tree.

**Figure 2.**
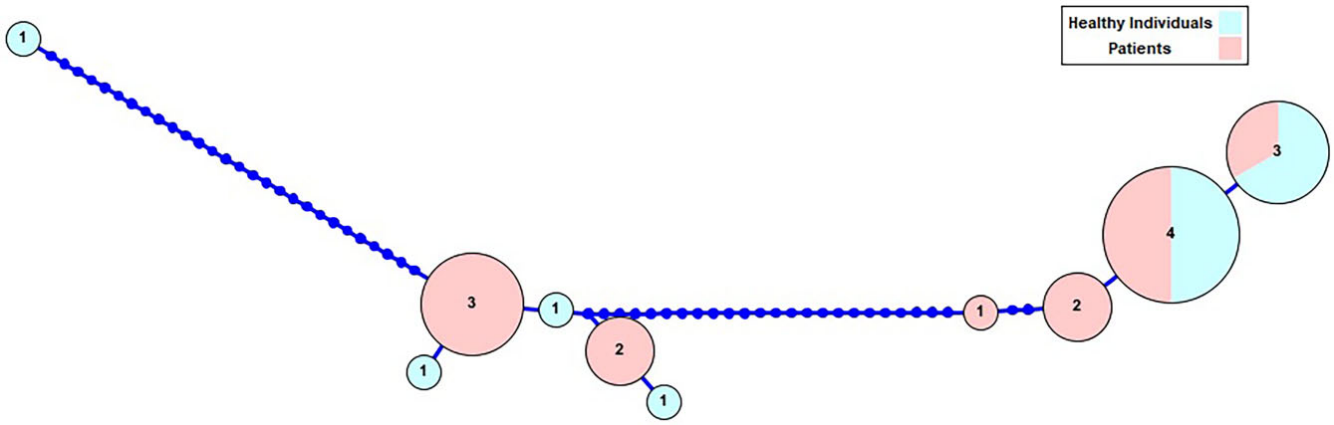
Haplotype network generated using ssrRNA nucleotide sequences of the *E. gingivalis* obtained in this study.

A toal of 19 of the 94 samples (20.2%) were positive for *E. gingivalis* by nested PCR (with Entam1/Entam2 and GEgFST1- GEgFST2/EgST1/2); 11 of these 19 positive samples came from the oral cavity of patients with dental disease and the other eight came from healthy individuals. The positivity rates were found to be (11/36) in patients with dental disease and 13.8% (8/58) in healthy individuals. All sequences were confirmed as *E. gingivalis* by the BLAST algorithm. Regarding the subtypes, 12 samples were identified as ST1 and 7 were ST2. The phylogenetic tree was constructed using 22 different sequences (19 of the sequences are detected in this study) (Fig. 1). In addition, all the obtained sequences were submitted to GenBank (Some accession numbers of sequences: OP456213, OP456215, OP456304, OP422447, OQ932783O, and Q932784). Additionally, the analysis of haplotypes was conducted to visualize of genetic diversity among the obtained sequences more clearly. The generated haplotype network presents in Fig. 2.

### The relationship between the presence of *E. gingivalis* and several characteristics of study groups

In this study, as an indicator of oral hygiene, tartar, periodontitis, smoking, tooth brushing habits, common tooth brush usage, mouthwash usage, drinking water preference, and presence of chronic diseases are the parameters analyzed. Clinical information and some lifestyle habits of *E. gingivalis* positive patients were given in Table 2. As a result, no significant relationships were found between the presence of *E. gingivalis* and patients’ demographics including age, gender, education, and income status (*P* > .05), and no significant correlations were observed between the presence of the parasite and the use of tap water, mouthwash, common toothbrushes, or tobacco (*P* > .05). However, among the *E. gingivalis*-positive individuals a significant association was observed between tobacco usage and dental diseases (*P* = .012). In addition, the presence of *E. gingivalis* was significantly higher in patients with dental diseases compared to healthy individuals (*P* = .02).

**Table 2.** Clinical information and some lifestyle habits of *E. gingivalis* positive patients.

Subject codes	Subtypes	Age	Gender	Educational status	Generalized periodontitis	Generalized gingivitis	Mild/localized gingivitis	Subgingival tartar	Supragingival tartar	Chronic diseases	Smoking	Mount wash usage	Common tooth brush	Tap water
P1	ST1	49	F	U	+	+		+	+	N	N	Y	N	Y
P2	ST1	35	M	FS		+				N	Y	N	N	N
P3	ST1	20	F	U	+	+		+	+	N	N	N	N	N
P4	ST1	55	M	FS	+	+		+	+	N	Y	N	N	N
P5	ST1	40	M	U	+	+		+	+	N	Y	N	N	N
P6	ST1	42	F	FS		+				N	N	N	N	N
P7	ST1	38	F	U		+		+	+	N	N	N	N	N
P8	ST2	22	F	FS	+	+		+	+	CD HS	Y	N	N	N
P9	ST2	33	M	FS	+	+		+		N	Y	N	N	Y
P10	ST2	44	M	FS		+		+		N	Y	N	N	N
P11	ST2	23	M	U			+	+		N	N	N	N	N

F: female, M: male, Y: yes, N: no, FS: first school, HS: high school, U: university, CD: cardiac disease, and HS: hypersensitivity.

Clinical information and some lifestyle habits of *E. gingivalis* positive healthy individuals were given in Table 3. Any relationship was found between the presence of *E. gingivalis* and healthy individuals’ demographics such as age, gender, education, and income status (*P* > .05). Additionally, parasite presence and using tap water, mouthwash, common tooth brush usage, and tobacco were not found associated (*P* > .05).

**Table 3.** Clinical information and some lifestyle habits of *E. gingivalis* positive healthy individuals.

Subject codes	Subtypes	Age	Gender	Educational status	Generalized periodontitis	Generalized gingivitis	Mild/localized gingivitis	Subgingival tartar	Supragingival tartar	Chronic diseases	Smoking	Mount wash usage	Common tooth brush	Tap water
H1	ST1	21	F	U	–	–	–	–	–	CD	N	N	N	N
H2	ST1	33	F	U	–	–	–	–	–	RA	N	N	N	Y
H3	ST1	20	F	U	–	–	–	–	–	N	N	N	N	N
H4	ST1	21	F	U	–	–	–	–	–	N	N	N	N	Y
H5	ST1	21	F	U	–	–	–	–	–	N	N	N	N	N
H6	ST2	21	F	U	–	–	–	–	–	N	N	N	N	N
H7	ST2	22	F	U	–	–	–	–	–	N	N	N	N	Y
H8	ST2	21	F	U	–	–	–	–	–	N	N	N	N	Y

F: female, M: male, Y: yes, N: no, U: university, CD: cardiac disease, and RA: rheumatoid arthritis.

## Discussion

A total of 19 participants (19/94, 20.2%) were found positive for *E. gingivalis* by using molecular techniques. In detail, the positivity rates of *E. gingivalis* were found to be 30.5% (11/36) in patients with dental disease and 13.8% (8/58) in healthy individuals. There is no significant association between demographics such as age, gender, educational status, income levels, and some lifestyle habits such as using tap water, mouthwash, and tobacco (*P* > .05). However, Arpağ and Kaya (2020) found a significant association between the presence of *E. gingivalis* and demographic data including gender, education status, frequency of dental visits, and brushing frequency (Arpağ and Kaya 2020). Additionally, another study reported a higher prevalence of *E. gingivalis* among individuals aged 45 years and above (Badri et al. 2021). Considering the age, the discrepancy in the results from different reports may be due to the difference in the average age of the participants. On the other hand, a significant correlation was found between the presence of *E. gingivalis* (30.5%, 11/36) and dental diseases (*P* = .02) in this study. Our findings align with previous studies that reported the detection of *E. gingivalis* in 29% and *T. tenax* in 2% of patients with gingivitis or periodontitis (Bardak et al. 1998). Similarly, another study investigating the density of *E. gingivalis* and *T. tenax* in microbial dental plaque found that 34.7% of the samples harbored *E. gingivalis*, while only 1.2% had *T. tenax* (Çeliksöz et al. 2001).

In the initial investigations conducted in the Eastern Anatolia area, the prevalence of *E. gingivalis* was determined to be 23.3% (Hakgüdener et al. 1998). Additionally, in another report involving 220 patients, the detection of *E. gingivalis* and/or *T. tenax* was found in 26.4% of the patients, with *E. gingivalis* identified in 21.8% of the cases and both *E. gingivalis* and *T. tenax* detected in 3.6% of the cases (Özçelık et al. 2010). In a study from the Aegean region, in 46 samples (33 with periodontal disease, 13 healthy subjects) in seven (19.44%) samples *E. gingivalis* was detected (Abualqomsaan et al. 2010). A previous study from Central Anatolia has reported that 60 (34.2%) out of 175 patients were found to be positive solely for *E. gingivalis* (Yazar et al. 2016). There was no presence of parasite around the healthy implants though out of 101 peri-implantitis lesions, 31 (30.7%) of them was found *E. gingivalis* positive. These results support the consistency of our findings concerning the presence and prevalence of *E. gingivalis* in patients with dental diseases and healthy participants.

At least two molecular subtypes of *E. gingivalis* have been described as ST1 and ST2 and both subtypes can be found in healthy people as commensal microorganisms (Garcia et al. 2018b, Rahdar et al. 2019, Bao et al. 2020). Although *E. gingivalis* is considered associated with periodontal diseases, subtypes are not shown to have any particular differences in demographics, geographic, or clinical properties (Stensvold et al. 2021).

In our study, the majority of the samples (12 out of 19) were categorized as ST1, while seven were identified as ST2. As in previous studies, no correlation was observed between the molecular subtypes and specific characteristics. According to a recent study, the frequency of detection of *E. gingivalis* (ST1 or ST2) in subgingival biofilm samples taken from periodontal pockets of patients was 88.3%, higher than previous studies using molecular methods. In addition, *E. gingivalis* was found to be significantly higher in pathological regions compared to healthy regions of control or periodontitis patients (Dubar et al. 2020). While one of the previous reports claimed that the prevalence of the ST2 subtype was significantly increased in the case of the patients undergoing orthodontic treatment (Garcia et al. 2018b), the ST2 variant was observed less frequently than ST1 in pathological regions (Dubar et al. 2020).

In conclusion, this study utilized molecular techniques to detect the presence of *E. gingivalis* in healthy individuals and patients with dental diseases, identifying two distinct subtypes (ST1 and ST2), marking the first study in Turkey to perform such molecular characterization. Further research is necessary with a larger sample size to determine the biological significance of these *E. gingivalis* subtypes.

## Consent to participate

An informed consent that explained the purposes, benefits, and risks of the study was signed by all participants.

## Data Availability

The datasets generated during and/or analyzed during the current study are available from the corresponding author on reasonable request.
